# Prof. E. M. Moore’s Surgical Clinic at the Buffalo General Hospital

**Published:** 1881-02

**Authors:** F. Peterson


### PROF. E. M. MOORE'S SURGICAL CLINIC AT THE BUFFALO GENERAL HOSPITAL.

JANUARY 5, 1881.

REPORTED BY F. PETERSON, M. D.

DR. B. BARTOW, at Prof. Moore's request, proceeded to show the students his method of treating spinal curvatures with the leather jacket. First, a plaster of Paris jacket is made as a mould for a cast of the trunk of patients, and about this cast the belt-leather, softened by water, is wound, hammered, moulded, and left to dry, until a light, perfectly-fitting jacket is made, having all the advantages of any other, besides cleanliness and endurance, in which plaster of Paris is deficient. The leather splint's weight is about three pounds, while that of plaster is six pounds. After this exposition by Dr. Bartow, the following patients were brought before the class:

CASE I.—A case of exsection of the elbow in a man of middle age. It is seven weeks since the operation. Flexion, pronation, and supination are good.

CASE II.—R. G. T., male; aged 27; home, Michigan; caries of right ischial tuberosity, caused by a fall two years before, from a wagon. It had opened and discharged soon after, and a fistula was formed between the scrotum and right thigh, about one-third of an inch in diameter. Ether having been administered, the fistula was slightly enlarged, the finger introduced, and a spiculum of loose bone felt in the vicinity of the thyroid foramen. With some difficulty, and by manipulation with forceps and chisel, the bone was dislodged and removed, and found to be the tuber ischii. A rubber drainage-tube of a half-inch calibre was inserted, not only for the purpose of allowing frequent antiseptic syringing and cell-proliferation from the bottom, but also to admit a finger for subsequent examinations.

Case III.—Male; aged 25; angry, suppurating, open bubo. This, the professor treats according to the plan and prescription of Ricord. To the class of phenols, all of which have powerful antiseptic properties, belong the spices and other carminatives. To the prescription of Ricord, found in the French Pharmacopeia, our Tinctura Lavandulæ Comp. comes nearest, and with this antiseptic liquid, the bubo is cleansed thrice daily. In addition, the sides of the bubo are held apart by wads of lint, and pressure with adhesive plaster. This case, having been thus treated for a week, already shows signs of marked improvement.

Case IV.—Male; aged 27; had chancre and gonorrhœa, and now has advanced bubo as a consequence. There are two over the left ligament of Poupart. Such communicate usually beneath the fascia of that region. If they are opened too early they become chronic. It is best to allow them to ripen. These are sufficiently mature for incision. He prefers to open them deeply, and at the lowest point, and he suited the action to his preference with the remark that "the way of the transgressor is hard." He always makes use of the director. About one ounce or two of pus and blood were removed.

Case V.—Male; aged 35; gangrene of fingers from freezing. Began the celebration of New Year's day by wandering drunk about the streets on its eve, and turned up shortly after in the hospital with his hands in this condition. The treatment had been warm applications and carbolized oil. Time is required, and the progress of desquamation and gangrene will be watched.

CASE VI.—Male; aged 28; both arms and face burned at the Birge factory fire, two weeks before. There are three classes of burns: the superficial, which does not destroy the skin; the superficial, destroying the skin; and the deep, which destroys any and everything. This patient belongs to the extensive and second superficial variety. It had been treated with carbolized oil. The professor thinks nothing supersedes at present the old-time mixture of lime-water and olive oil, used at the Carron Iron Works, and the gentle application about the burn of cotton. The Carron oil forms a soft soap, like mucus, and its soothing action no doubt depends upon the exclusion of air from the irritated nerve extremities. This case will require skin-grafting.

JANUARY 19, 1881.

CASE I.—Female; aged 9; patient of Dr. Mynter; an abdominal tumor in umbilical region. It was supposed by some that the spleen is enlarged and that the patient is suffering from leucocythemia, but microscopic examination failed to strengthen the diagnosis. The tumor is exceedingly hard, circumscribed, movable in the abdomen, and gives a resonance transmitted from the intestines beneath it proves it mesenteric. As the patient is too young to have malignant disease, the Professor thinks it one of those remarkable and rare cases of a plate of cartilaginous or osseous material, forming in the mesentery. Of course, no therapeutic aid can be rendered.

CASE II.—Male child; aged 2½ years; had the following deformities when born: Right hand has a perfect thumb, and one wide finger having the phalanges of two perfect fingers within it; left foot has three toes only, the large one of great size; the right foot is a case of valgus, and the lower third of the leg is bent forwards at an oblique angle. Infant walks on the inner side of the foot and the leg is shorter than the other. In order to remedy this unsightly distortion, the Professor divided the tendons of the peronei and the great tendon of the soleus and gastrocnemius, the patient being under ether. To straighten the leg, a valvular incision was made over the projecting angle of the tibia, the bone broken by manipulation, and portions of bone removed from the extremities. Adhesive plaster, some absorbent cotton and a bandage, were then applied. In a short time plaster of Paris bandages will be required to hold the limb in position. They are best, too, because an opening can be made over the wound in the leg to give egress to pus, and the bones still remain inflexible.

Case III.—Male; aged 30; carpenter. Two years ago he thrust a chisel into the palm of his right hand, just over the flexor of the index finger. The theca of the tendon became immediately inflamed and spread up the whole finger. The physician in attendance drew out the tendon at the palmar opening. Since then he has been unable to flex the finger, it remaining strictly extended and interfering greatly with his work as a carpenter. He desires amputation between the first and second phalanges. The doctor amputated the member as he wished, remarking at the same time that it would be better to remove the whole finger and the head of the metacarpal bone also, thus substituting by approximation the middle for the index finger. In the foot as much of the metatarsal bones as possible is to be allowed to remain, as it forms the ball of the foot, but it is necessary to make the hand compact and prehensile.

